# Dry Beriberi Manifesting as Acute Inflammatory Demyelinating Polyneuropathy in a Patient With Decompensated Alcohol-Induced Cirrhosis

**DOI:** 10.7759/cureus.11281

**Published:** 2020-10-31

**Authors:** Amaninder Dhaliwal, Jessica L Larson, Banreet S Dhindsa, Neil Bhogal, Fedja A Rochling

**Affiliations:** 1 Gastroenterology and Hepatology, University of South Florida Morsani College of Medicine, Tampa, USA; 2 Internal Medicine, University of Nebraska Medical Center, Omaha, USA; 3 Gastroenterology and Hepatology, University of Nebraska Medical Center, Omaha, USA

**Keywords:** case report, beri beri, alcohol related cirrhosis, polyneuropathy

## Abstract

Thiamine (vitamin B1) deficiency is uncommon in developed countries and is most commonly seen in patients with poor dietary intake, malabsorption syndromes, and alcoholism. With the increasing rates of alcohol use, thiamine deficiency is likely an under-recognized and potentially reversible cause of sensorimotor dysfunction called dry beriberi. We present a case of profound lower extremity weakness in a 28-year-old female from Nepal with decompensated alcohol-induced cirrhosis. Based on laboratory testing, it was determined that the cause of her neuropathy was dry beriberi. She was subsequently started on thiamine replacement therapy with slow improvement over the next six months.

## Introduction

Dry beriberi is a nutritional neuropathy that results from thiamine (vitamin B1) deficiency and is most commonly seen in individuals with a poor diet, alcoholism, or malabsorption syndromes. Thiamine is a cofactor that is essential for maintaining the myelin sheaths of neurons; however, the human body’s storage only lasts up to 18 days. Dry beriberi commonly manifests as a symmetric sensorimotor neuropathy that primarily affects the distal extremities. In the majority of cases, it appears as a gradual, insidious onset but occasionally can appear rapidly resembling an acute inflammatory demyelinating polyneuropathy (AIDP). Dry beriberi is uncommon in developed countries, but its incidence is increasing alongside the growing prevalence of alcohol use. We present a case of dry beriberi manifesting as AIDP in a patient with decompensated alcohol-induced cirrhosis.

## Case presentation

A 28-year-old female from Nepal who recently immigrated to the United States presented to the ED with complaints of abdominal distention, fatigue, and six weeks of progressive bilateral lower extremity weakness. She was recently discharged from an outside hospital with a new diagnosis of decompensated liver cirrhosis with ascites. She reported drinking homemade alcoholic beverages daily while in Nepal but was unable to quantify the amount, given inconsistent history due to language and cultural barrier. She was taking thiamine 100 mg oral daily, which was not continued on admission. On evaluation, she was extremely malnourished with a BMI of 14.6 due to lack of appetite and a diet consisting mostly of rice and beef broth soup. Physical exam demonstrated scleral icterus, abdominal distention with tenderness in the right upper quadrant, hepatosplenomegaly, and jaundice. Laboratory studies were notable for platelet count of 96,000/uL, albumin 2.8 g/dL, aspartate aminotransferase (AST) 52 U/L, alanine aminotransferase (ALT) 30 U/L, total bilirubin 34.5 mg/dL, international normalized ratio (INR) 1.8. Her initial model for end-stage liver disease (MELD) score was 26, and she was evaluated for liver transplantation. Other causes of cirrhosis, including viral, hemochromatosis, autoimmune, Wilson’s disease, primary biliary cirrhosis, and primary sclerosing cholangitis, were excluded, and it was determined that she had alcohol-induced cirrhosis. Due to her extreme malnutrition and weakness, she was listed inactive on the transplant list.

The patient had bilateral lower extremity weakness and paresthesias preventing her from standing unassisted. She also demonstrated areflexia, tenderness to palpation, and pain with movement of lower extremities. Initial workup for weakness included vitamin B12, folate, and vitamin B6 levels. Vitamin B12 and folate returned within normal limits, but she was found to be deficient in vitamin B6 at 11.7 nmol/L and was started on replacement therapy. After 10 days of supplemental vitamin B6 and physical therapy, she had not improved, and neurology was consulted. An electromyogram was performed and consistent with severe sensorimotor axonal polyneuropathy with active denervation. Cerebrospinal fluid (CSF) analysis was also recommended and showed a slightly elevated protein at 47 mg/dL but not elevated enough to be consistent with Guillain-Barré syndrome (GBS). Additional labs were drawn, and she was found to have a low thiamine level at 55 nmol/L (normal: 70-80 nmol/L). She was started on replacement therapy with slow improvement. By six months of aggressive thiamine replacement, she could walk 200 feet with a walker and stand without assistance.

## Discussion

Thiamine is a water-soluble vitamin that is found in a variety of whole grains, nuts, and legumes. In the western world, many of our cereals and white grains are now fortified with thiamine making its deficiency uncommon. However, several populations are at risk of becoming thiamine deficient, including individuals with a poor diet, alcoholism, long-term parenteral nutrition use, or malabsorption syndromes.

Thiamine deficiency typically manifests as either Wernicke-Korsakoff syndrome or beriberi. Beriberi has historically been divided into two separate types: wet beriberi and dry beriberi. Wet beriberi affects the cardiovascular system, whereas dry beriberi impairs the central and peripheral nervous systems.

Both Wernicke-Korsakoff syndrome and dry beriberi affect the nervous system. This may be due to thiamine’s use as a cofactor in the metabolism of carbohydrates to form adenosine triphosphate (ATP), generation of the neurotransmitter acetylcholine, and formation of myelin sheaths around axons [1]. Currently, there are three ways by which chronic alcohol intake can lead to thiamine deficiency. First, people with alcoholism can have a poor dietary intake of nutritious foods. Second, alcohol inhibits the absorption of thiamine across the gastrointestinal tract [2]. Lastly, alcohol leads to decreased utilization of thiamine in cells. Because only a small amount of thiamine is stored in the liver, a continuous supply of thiamine from the diet is needed. With limited tissue stores and inadequate nutrition, thiamine can be depleted in as little as 18 days [3].

Dry beriberi commonly develops as a gradual, insidious sensorimotor neuropathy in the distal extremities symmetrically. It can easily be confused with other types of neuropathy, including alcoholic neuropathy and AIDP, also known as GBS [4]. Our patient had a history of alcohol abuse and subsequently developed cirrhosis. She was extremely malnourished and underweight. She acutely developed profound weakness, areflexia, and paresthesia of lower extremities consistent with GBS. CSF analysis, however, was not consistent with GBS. 

There have been several other case reports of dry beriberi resembling GBS [5-7] and even fewer case reports of patients with liver disease and concurrent dry beriberi [8,9]. Our case demonstrates the importance of having a high index of suspicion for thiamine deficiency in patients with alcohol-induced cirrhosis. By testing our patient’s serum vitamin B1 level, the cause of her acute decline was discovered, and subsequently, she was started on thiamine replacement therapy. Had her thiamine level been tested sooner, she would have avoided unnecessary tests and shortened the length of her hospital stay. These patients show early response and can take up to six months for a full recovery, which is, unfortunately, not seen in all patients [10].

## Conclusions

With the increasing rates of alcohol use, dry beriberi represents a potentially underdiagnosed cause of neuropathy. It is important to consider this diagnosis early in the evaluation of patients with history of excessive alcohol use who presents with symmetric sensorimotor neuropathy, as early replacement can prevent progression and potentially reverse neurological manifestations. Response can be variable and some patients can have irreversible damage.
